# Effect of glass markings on drinking rate in social alcohol drinkers

**DOI:** 10.1093/eurpub/ckw142

**Published:** 2016-10-20

**Authors:** David M. Troy, Angela S. Attwood, Olivia M. Maynard, Nicholas E. Scott-Samuel, Matthew Hickman, Theresa M. Marteau, Marcus R. Munafò

**Affiliations:** 1 MRC Integrative Epidemiology Unit (IEU), University of Bristol, Bristol, UK; 2 UK Centre for Tobacco and Alcohol Studies, School of Experimental Psychology, University of Bristol, Bristol, UK; 3 School of Experimental Psychology, University of Bristol, Bristol, UK; 4 School of Social and Community Medicine, University of Bristol, Bristol, UK; 5 Behaviour and Health Research Unit, Institute of Public Health, University of Cambridge, Cambridge, UK

## Abstract

**Background:** The main aim of these studies was to explore the influence of volume information on glassware on the time taken to consume an alcoholic beverage. **Methods:** In Study 1, male and female social alcohol consumers (*n* = 159) were randomised to drink 12 fl oz of either low or standard strength lager, from either a curved glass marked with yellow tape at the midpoint or an unmarked curved glass, in a between-subjects design. In Study 2, male and female social alcohol consumers (*n* = 160) were randomised to drink 12 fl oz of standard strength lager from either a curved glass marked with ¼, ½ and ¾ volume points or an unmarked curved glass, in a between-subjects design. The primary outcome measure for both studies was total drinking time of an alcoholic beverage. **Results:** In Study 1, after removing outliers, total drinking time was slower from the glass with midpoint volume marking [mean drinking times (min): 9.98 (marked) vs. 9.55 (unmarked), mean difference = 0.42, 95% CI: −0.90, 1.44]. In Study 2, after removing outliers, total drinking time was slower from the glass with multiple volume marks [mean drinking times: 10.34 (marked) vs. 9.11 (unmarked), mean difference = 1.24, 95% CI: −0.11, 2.59]. However, in both studies confidence intervals were wide and also consistent with faster consumption from marked glasses. **Conclusion:** Consumption of an alcoholic beverage may be slower when served in glasses with volume information. Replication in larger studies is warranted.

## Introduction

Excessive alcohol consumption is a major public health problem.^1^^,^^2^ Worldwide, approximately 16% of drinkers aged 15 years or older engage in heavy episodic drinking (defined as consuming 60 or more grams of pure alcohol on at least one occasion at least monthly).^1^ The health costs of heavy consumption are substantial with alcohol being the world’s third leading cause of ill health and premature death.^3^ Therefore, the need for a comprehensive toolkit to reduce alcohol consumption is pressing; especially while other effective population level interventions to reduce alcohol consumption are delayed—such as increasing price^4–7^ and reducing availability.^8^

Choice architecture interventions have become popular among policy makers in recent years^9^^,^^10^ because they are low cost and do not require legislation at a national level. These interventions alter the way choices are presented, and/or the properties or placement of objects or stimuli within a micro-environment in an attempt to prompt healthier behaviours. Interventions of this type do not coerce or prohibit any action on the part of the individual (see Ref. 11 for an operational definition). By altering the environments within which people make choices, choice architecture interventions allow behaviour to be influenced at the population level.^11^ The advantages of these interventions are that they mainly rely on automatic psychological processes,^12–14^ resulting in an impact regardless of individual differences. Choice architecture interventions which can be embedded within micro-environments where alcohol consumption occurs (e.g. public houses and home environments) are likely to be particularly effective, given the wide potential reach of such interventions.

Glassware is one potential target for choice architecture interventions. It has been shown that the shape of glassware affects how individuals interact with it. For example, when asked to pour a standard measure, students and bartenders poured more alcohol into short, wide glasses than tall, slender glasses.^15^ A mechanism to explain this difference is that individuals tend to focus their attention on the height the liquid reaches and insufficiently compensate for the width of the glass.^16^ People also tend to estimate that tall, slender glasses hold more liquid than wide glasses of the same volume.^17^^,^^18^ This may increase actual consumption volume while reducing perceived consumption volume.^18^ In support, a study investigating the effect of glass shape on drinking rate^19^ reported that beer (but not lemonade) was consumed slower from a straight glass compared with a curved glass. In addition, participants judged glass midpoint in a separate computerised task, and there was evidence of a positive relationship between degree of error and total drinking time. This suggests that inaccuracies in volume judgements may be greater when changes in height and volume of liquid are not directly proportional, which may lead to faster drinking speed and/or greater overall intake. Therefore, providing volume information on glassware may mitigate this effect by rectifying these perceptual errors. Providing visual cues to inform consumptive behaviour has shown promise in other areas; food researchers have shown that plates containing portion size information induce greater weight loss in obese patients compared with plates without portion size information.^20^^,^^21^

Drinkers appear to self-titrate their alcohol intake under *ad libitum* conditions, albeit imperfectly, to achieve similar blood alcohol levels when consuming drinks of differing strengths.^22–25^ Under laboratory conditions, there is evidence that drinkers alter their drinking rate when consuming lagers of different alcohol-by-volume profiles.^26^ Therefore, it would seem important to evaluate the effect of volume information on lagers of different strength.

We investigated whether volume information on curved glasses alters the time taken to consume an alcoholic beverage. In addition, in Study 1 we investigated dose effects by including a lager strength factor. We hypothesised that curved glasses with volume information would result in longer drinking times compared with curved glasses with no volume information. We also hypothesised that glass markings would slow drinking times more for standard strength lager compared with low strength lager.

## Study 1

### Methods

#### Design and overview

The study used a 2 × 2 design with glass marking (unmarked, marked) and lager strength (low, standard) as predictor variables. In order to maintain equal numbers of participants in each condition, we randomly assigned 50% of participant numbers to the markings condition and 50% to the no markings condition. This ensured that overall there were equal numbers of participants in both conditions. We did this separately for male and female participants to ensure an equal sex ratio across groups. Group allocation was randomised via randomisation software (https://www.randomizer.org). Data were captured by video recording and the primary outcome measure was total drinking time. Subjective measures of alcohol craving and mood were taken to assess any changes during the study session. Ethics approval was obtained from the Faculty of Science Research Ethics Committee at the University of Bristol (reference: 310108288). The study was conducted according to the revised Declaration of Helsinki (2000) and Good Clinical Practice guidelines (fifth revision). Written informed consent was obtained from all participants.

#### Participants

Social alcohol drinkers, aged between 18 and 40 years and who reported consuming between 10 and 50 units/week if male, or between 5 and 35 units/week if female, were recruited from the staff and students of the University of Bristol, and from the general population by means of poster and flyer advertisements, existing participant database and word-of-mouth. Exclusion criteria included current use of illicit substances (excluding cannabis), current use of psychiatric medication, a strong family history of alcoholism (defined as at least one first-degree relative or two or more second-degree relatives) and not drinking/liking lager. Eligibility was ascertained by self-report. Participants were asked to abstain from alcohol consumption for 12 h prior to the test session, and were only enrolled onto the study if they provided a 0 µg/100 ml breath alcohol reading. Participants were reimbursed £7 or awarded course credit, as appropriate, at the end of the study.

#### Materials

**Figure 1:**
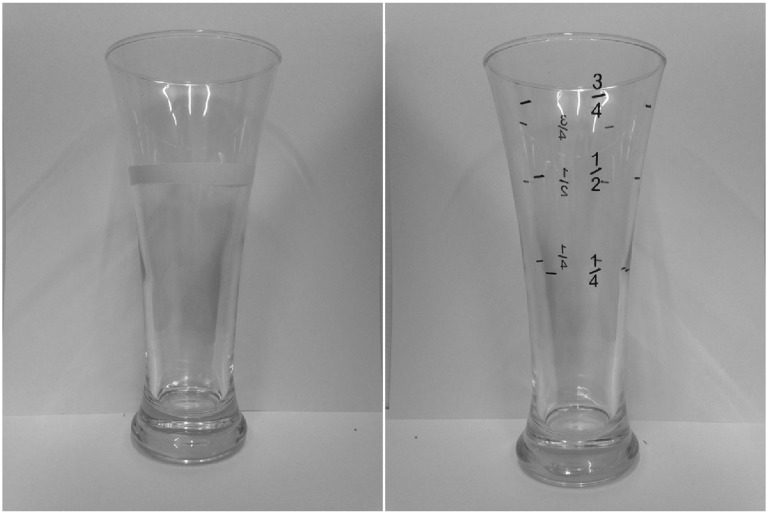
Marked glasses used in Studies One (left) and Two (right). Both curved glasses have 12 fl oz volume capacity

Alcoholic beverages used were low strength (Bière des Moulins^TM^, 3.8% ABV) and standard strength (St. Cervois^TM^, 4.8% ABV) lagers. Glass type used was a curved beer glass (volume: 12 fl oz) as used in our previous study.^19^ One glass had the midpoint marked with a band of yellow tape (figure 1). The other glass remained unmarked.

Questionnaire measures comprised the Alcohol Use Disorders Identification Task (AUDIT)^27^ to examine hazardous/harmful drinking and risk of dependence and the Alcohol Urges Questionnaire (AUQ)^28^ to assess craving for alcohol. Visual analogue scales (VASs) from 0 to 100 of six moods (happiness, drowsiness, depression, anxiety, energy and irritability) and alcohol craving were also administered.

#### Procedure

Experimental sessions lasted approximately 30–45 min and all testing took place between 12:00 and 18:00 hours. Upon arrival, participants were given an opportunity to read the information sheet and ask questions before providing written informed consent. Participants were told that the study examined the effects of alcohol consumption on word search performance, in order to disguise the primary outcome measure. Participants were screened to ensure that they met the inclusion criteria, and breath alcohol was measured.

In the main session, participants received 12 fl oz (i.e. a full glass) of lager (either low or standard strength in an unmarked or marked glass as per randomisation). Drinks were chilled prior to serving and were poured immediately prior to consumption in order to ensure that carbonation was consistent across participants. Self-report measures of alcohol use (AUDIT), alcohol craving (AUQ, VAS) and mood (VASs) were obtained. Participants were told that they should consume all of the drink at their own pace whilst watching a nature documentary (Earth: The Journey of a Lifetime, BBC Worldwide 2008). The experimenter started the film (at the same point for all participants) and left the room. The drinking session was recorded using a video camera (Hitachi Hybrid Camcorder DZHS500E). Participants opened the door when they had finished their beverage, the experimenter returned and presented participants with a word-search task in which they were instructed to find as many words as possible in 4 min. This was intended to disguise the nature of the study, and these data were not analysed. Then, measures of alcohol craving (AUQ, VAS) and mood (VASs) were administered again. Finally, participants were informed that debriefing information would follow via email at the end of the study, and were reimbursed.

### Statistical analysis

The video recordings were viewed by one researcher, and total drink time (i.e. time from initiation of first sip to end of last sip) was extracted. To assess extraction reliability, 20% of videos were randomly chosen and assessed by a second independent rater. Inter-rater reliability was high (intraclass correlation = 0.99). Total drinking time outliers were identified using boxplots, and defined as 1.5 times the interquartile range above quartile 3 or below quartile 1. We identified outliers in order to exclude participants who may not have been drinking naturalistically in the laboratory environment, in order to thereby capture a more natural range of drinking times as has been carried out in other *ad libitum* drinking studies.^24^^,^^25^

Total drinking time data were analysed using multiple regression including glass marking (unmarked, marked) and lager strength (low, standard) as predictor variables and an interaction term of glass marking and lager strength. For the analysis of the AUQ and VAS data, linear regressions were carried out with glass marking (unmarked, marked) as the predictor. Analyses were conducted using IBM SPSS (SPSS Statistics Software Release 21, IBM Corporation).

A previous study^19^ found an effect size of *d* = 0.91 when measuring the difference in drinking times between straight and curved glasses. In order to be conservative we powered our study to detect a smaller effect size of *d* = 0.45. We calculated that 158 participants would be required to provide 80% power at an alpha level of 5% in order to detect this effect. In total, 160 participants were recruited to allow equal allocation to the four experimental conditions.

The data that form the basis of the results presented here are available from the University of Bristol Research Data Repository (http://data.bris.ac.uk/data/), doi: 10.5523/bris.gujajy0f45po11lz1dln554f4.

### Results

Participants (*n* = 159; 80 female) were on average aged 22 years (SD* = *4, range 18–39), had a body mass index (BMI) of 23 kg/m^2^ (SD = 3, range 18–39) and had an AUDIT score of 10 (SD = 4, range 2–27). Participant characteristics are shown in table 1. One female was randomised to the marked condition in error, and data from one male participant were unusable due to video recording malfunction. An age value for one participant was not recorded during data collection. One missing questionnaire response was inserted based on the median of the sample for that specific question.

**Table 1:** Characteristics of participants

	Study 1		Study 2	
	Marked (*n* = 80)	Unmarked (*n* = 79)	Marked (*n* = 80)	Unmarked (*n* = 80)
Sex (female)	41 (51%)	40 (51%)	40 (50%)	40 (50%)
Age (years)	22 (4)	22 (4)	20 (3)	22 (4)
BMI (kg/m^2^)	23 (3)	22 (2)	23 (3)	23 (3)
AUDIT	10 (4)	10 (4)	10 (3)	10 (4)

Notes: Standard deviation is shown in parentheses for continuous measures. BMI, body mass index; AUDIT, Alcohol Use Disorder Identification Task.

There was no evidence of an interaction between glass marking and lager strength (mean difference = 1.68, 95% CI: −0.95, 4.32) after removing three outliers and the interaction term was not included in subsequent analyses. On average, participants consumed their drink more slowly from marked glasses than from unmarked glasses (mean difference = 0.42, 95% CI: −0.90, 1.74) and consumed standard strength lager faster compared with low strength lager (mean difference = −0.55, 95% CI: −1.87, 0.77) after removing outliers. When lager strength and outliers were removed, participants on average consumed their drink more slowly from marked glass compared with unmarked glasses (table 2). There was no evidence that glass marking was associated with post-consumption alcohol craving (AUQ, VAS) or mood (VAS) (table S1 in Supplementary data).

**Table 2:** Effect of glass markings on total drinking time

	Mean drinking time (min)			
	Marked	Unmarked	Mean difference	95% CI
Study 1				
Full sample (*n* = 159)	10.37	9.90	0.47	−1.12 to 2.06
Outliers excluded (*n* = 156)	9.98	9.55	0.43	−0.89 to 1.75
Study 2				
Full sample (*n* = 160)	10.49	9.83	0.67	−0.91 to 2.25
Outliers excluded (*n* = 156)	10.34	9.11	1.24	−0.11 to 2.59

Note: Outliers defined as 1.5 times the interquartile range above quartile 3 or below quartile 1.

## Study 2

### Methods

#### Design and overview

We hypothesised that the very limited, if any, marking effects on drinking rate in Study 1 may have been due to insufficient markings (i.e. no quantitative volume information and only one marking). Therefore, Study 2 investigated whether more detailed volume information, with volume markings at ¼, ½ and ¾ points, would slow drinking time. This study was a between-subjects design with glass marking (unmarked, marked) as the predictor. As there was no clear evidence to suggest lager strength was associated with drinking time in Study 1, this condition was not included in Study 2. Ethics approval was granted by the Faculty of Science Research Ethics Committee at the University of Bristol (reference: 25091410961) and the study was conducted in accordance with the Declaration of Helsinki (2013) and Good Clinical Practice guidelines (sixth revision). Written informed consent was obtained from all participants. The protocol was registered at http://osf.io/946q2 prior to data collection.

#### Participants

Identical criteria were used to select participants as in Study 1, with an additional exclusion criterion of not having taken part in the previous study.

#### Materials

Study 2 used the same glasses as Study 1, but with explicit quantitative volume markings consisting of black adhesive stickers at ¼, ½ and ¾ volume points replacing the yellow tape on the marked glass in Study 1 (figure 1). Lager consumed was 5% ABV Grolsch^TM^. Mood VASs were replaced by the Positive and Negative Affect Schedule (PANAS).^29^ The alcohol craving VAS from Study 1 was not used, as it was felt the AUQ sufficiently captured alcohol craving. All other measures were identical to Study 1.

#### Procedure

The procedure for Study 2 was identical to Study 1, except the required period of abstaining from alcohol prior to the study was increased to 24 h to avoid potential hangover effects.

### Statistical analysis

Video recording of participants was identical to Study 1. For Study 2, additional topography measures were extracted by the lead researcher including total sip duration (i.e. time sipping), total interval duration (i.e. time between sips) and total number of sips taken, using a specifically designed MATLAB program (MATLAB and Statistics Toolbox: Release 2013a; The Mathworks, Inc.), which required a button press each time a sip was initiated (defined as liquid touching lips) and ended (defined as liquid leaving lips). This enabled secondary analysis of drink pattern, which may inform future research and interventions. Video analysis reliability for all drinking measures was assessed as in Study 1. Inter-rater reliability was high (*r*’s > 0.96).

Total drinking time data were analysed using linear regression with glass marking (unmarked, marked) as the predictor variable. Total sip duration, total interval duration and total number of sips were analysed as secondary outcomes. For the analysis of the AUQ and PANAS data, linear regressions were carried out with glass marking (unmarked, marked) as the predictor. Analyses were conducted using IBM SPSS (SPSS Statistics Software Release 21, IBM Corporation). The sample size calculation and outlier detection were the same as for Study 1.

The data that form the basis of the results presented here are available from the University of Bristol Research Data Repository (http://data.bris.ac.uk/data/), doi: 10.5523/bris.9p8s50hw70x61kgxrbunjesj5.

### Results

Participants (*n* = 160; 80 female) were on average aged 23 years (SD = 4, range 18–40), had a BMI of 23 kg/m^2^ (SD = 3, range 17–36) and had an AUDIT score of 10 (SD = 4, range 3–22). Participant characteristics are detailed in table 1. One missing questionnaire response was inserted based on the median for that specific question. Four outliers were removed.

On average, participants consumed their drink more slowly from marked glasses than from unmarked glasses (mean difference = 1.24, 95% CI: −0.11, 2.59) when four outliers were removed (table 2). A similar pattern of results was observed for total interval durations (mean difference = 1.27, 95% CI: −0.06, 2.61) when outliers were removed, with longer interval durations when participants consumed their drink from marked glasses compared with unmarked glasses. There was no evidence that glass marking was associated with total sip duration or total number of sips, and no evidence that glass marking was associated with post-consumption alcohol craving (AUQ) or positive or negative affect (PANAS) (table S1 in Supplementary data).

## Discussion

Our data suggest that providing implicit midpoint volume information alone appears to have minimal, if any, slowing influence on the drinking time of an alcoholic beverage, but providing more detailed volume markings at ¼, ½ and ¾ points may increase this influence. However, these results should be interpreted with caution as the confidence intervals around the estimated mean differences were wide and were also consistent with faster consumption from marked glasses. It should also be noted that removing outliers increased the mean difference in drinking times in Study 2. Together, findings from both studies provide tentative support for the hypothesis that volume information results in slower consumption of an alcoholic beverage.

One possible explanation for this effect is that individuals use volume perceptions to titrate their drinking rate of an alcoholic beverage, in order to control level of intoxication. Longer intervals between sips in the marked glass group in Study 2 would support this interpretation. It may be the case that providing volume information assists a drinker to more accurately gauge how much they have consumed, as perceived consumption reportedly affects subsequent behaviour.^18^ Explicit quantitative information, as used in Study 2, appears to have assisted drinkers to more effectively slow their drinking times compared with the marking used in Study 1.

These results have policy implications if the effects seen are replicated in larger studies. In the UK, the 2003 Licensing Act^30^ afforded powers to local licensing authorities to issue alcohol licences and enforce the conditions of the licence in their area. A local licensing authority would be able to add a requirement to stock glasses with volume information to its ‘menu’ of licensing conditions, which premises must accept in order to be granted a license or have a license renewed. Future studies should investigate applying volume markings to glassware in naturalistic settings (e.g. bars).

There are limitations to these studies that should be considered when interpreting the results. First, while we observed slower drinking times in marked glasses, it is unclear whether this would translate to reduced intake overall, as unit bias^31^^,^^32^ and personal consumption norms^33^ need to be overcome to reduce overall intake. To address this limitation, future studies should focus on comparing within-subject *ad libitum* consumption of multiple alcoholic drinks from marked and unmarked glasses across multiple sessions to determine whether overall intake can be reduced. Second, the volume marking in Study 1 may have failed to inform drinkers that it constituted the volume midpoint of the glass due to no explicit information denoting that fact communicated to participants; however, this was addressed in Study 2. Third, participants may have been unable to detect the difference (1% ABV) in the strength of the lagers in Study 1. Fourth, the differences observed within and between both studies could be explained by demand characteristics rather than experimental manipulation. Markings in the intervention conditions may have primed awareness in participants that their drinking behaviour was being monitored and this resulted in slower drinking times. Recent findings^34^ from a review on eating behaviour would support this interpretation, although the evidence is mixed for drinking behaviour.^35^ Fifth, both studies lacked statistical power to detect small effects that might still be relevant at a population level. Sixth, our sample comprised predominantly of undergraduate students and findings may not generalise to other consumer groups with different patterns of drinking.

In conclusion, our data provide tentative support that volume information may influence the rate of consumption of alcoholic beverages, but this may depend on the design and content of the information. An effect of glass markings could be due to volume information enabling more accurate volume judgements, which in turn could lead to slower consumption. Although our findings are ambiguous, there is sufficient evidence to warrant a larger study in the future. Further research is also needed to examine the mechanisms underlying the effects observed. If glass marking can be shown to influence rate of alcohol consumption, this has the potential to be implemented as one of a suite of viable choice architecture interventions in drinking environments in order to slow consumption rates and reduce alcohol-related harms.

## Supplementary Material

Supplementary Data

## References

[1] World Health Organisation. Global Status Report on Alcohol and Health, Geneva, Switzerland: World Health Organisation, 2014 Available at: http://apps.who.int/iris/bitstream/10665/112736/1/9789240692763_eng.pdf?ua=1 (22 July 2016, date last accessed).

[2] British Medical Association. Alcohol Misuse Tackling the UK Epidemic, London, UK: British Medical Association, 2008 Available at: http://www.dldocs.stir.ac.uk/documents/Alcoholmisuse.pdf (22 July 2016, date last accessed).

[3] World Health Organisation. European Action Plan to Reduce the Harmful Use of Alcohol 2012–2020, Geneva, Switzerland: World Health Organisation, 2012 Available at: http://www.euro.who.int/__data/assets/pdf_file/0008/178163/E96726.pdf?ua=1 (22 July 2016, date last accessed).

[4] Huang C Econometric Models of Alcohol Demand in the United Kingdom. London: HM Revenue & Customs, 2003.

[5] Patra J, Giesbrecht N, Rehm J, Bekmuradov D Are alcohol prices and taxes an evidence-based approach to reducing alcohol-related harm and promoting public health and safety—a literature review. Contemp Drug Probs 2012;39:7.

[6] Wagenaar AC, Tobler AL, Komro KA Effects of alcohol tax and price policies on morbidity and mortality: a systematic review. Am J Public Health 2010;100:2270–8. 20864710 10.2105/AJPH.2009.186007 PMC2951962

[7] Holmes J, Meng Y, Meier PS, Effects of minimum unit pricing for alcohol on different income and socioeconomic groups: a modelling study. Lancet 2014;383:1655–64. 24522180 10.1016/S0140-6736(13)62417-4 PMC4018486

[8] Room R, Babor T, Rehm J Alcohol and public health. Lancet 2005;365:519–30. 15705462 10.1016/S0140-6736(05)17870-2

[9] Department of Health. Healthy Lives, Healthy People: Our Strategy for Public Health in England, London, UK: Department of Health, 2010 Available at: https://www.gov.uk/government/uploads/system/uploads/attachment_data/file/216096/dh_127424.pdf (27 July 2016, date last accessed).

[10] Cabinet Office Behavioural Insights Team. Applying Behavioural Insight to Health, London, UK: Cabinet Office Behavioural Insights Team, 2010 Available at: https://www.gov.uk/government/uploads/system/uploads/attachment_data/file/60524/403936_BehaviouralInsight_acc.pdf (22 July 2016, date last accessed).

[11] Hollands G, Shemilt I, Marteau T, Altering micro-environments to change population health behaviour: towards an evidence base for choice architecture interventions. BMC Public Health 2013;13:1218. 24359583 10.1186/1471-2458-13-1218 PMC3881502

[12] Hofmann W, Friese M, Wiers RW Impulsive versus reflective influences on health behavior: a theoretical framework and empirical review. Health Psychol Rev 2008;2:111–37.

[13] Marteau TM, Ogilvie D, Roland M, Judging nudging: can nudging improve population health? BMJ 2011;342:d228. 21266441 10.1136/bmj.d228

[14] Marteau TM, Hollands GJ, Fletcher PC Changing human behavior to prevent disease: the importance of targeting automatic processes. Science 2012;337:1492–5. 22997327 10.1126/science.1226918

[15] Wansink B, van Ittersum K Shape of glass and amount of alcohol poured: comparative study of effect of practice and concentration. BMJ 2005;331:1512–4. 16373735 10.1136/bmj.331.7531.1512 PMC1322248

[16] Wansink B, van Ittersum K Bottoms up! the influence of elongation on pouring and consumption volume. J Consum Res 2003;30:455–63.

[17] Piaget J The Mechanisms of Perception. London: Routledge & Kegan, 1969.

[18] Raghubir P, Krishna A Vital dimensions in volume perception: can the eye fool the stomach? J Marketing Res 1999;36:313–26.

[19] Attwood AS, Scott-Samuel NE, Stothart G, Munafò MR Glass shape influences consumption rate for alcoholic beverages. PLoS One 2012;7:e43007. 22912776 10.1371/journal.pone.0043007 PMC3422221

[20] Pedersen SD, Kang J, Kline GA Portion control plate for weight loss in obese patients with type 2 diabetes mellitus: a controlled clinical trial. Arch Intern Med 2007;167:1277–83. 17592101 10.1001/archinte.167.12.1277

[21] Kesman R, Ebbert J, Harris K, Schroeder D Portion control for the treatment of obesity in the primary care setting. BMC Res Notes 2011;4:346. 21906302 10.1186/1756-0500-4-346 PMC3197506

[22] Shortt RG, Vogelsprott MD Social drinkers self-regulation of alcohol intake. J Stud Alcohol 1978;39:1290–3. 703328 10.15288/jsa.1978.39.1290

[23] Van Houten R, Van Houten J, Malenfant JL The effects of low alcohol beverages on alcohol consumption and impairment. Behav Modif 1994;18:505–13. 7980376 10.1177/01454455940184007

[24] Geller ES, Kalsher MJ, Clarke SW Beer versus mixed-drink consumption at fraternity parties—a time and place for low-alcohol alternatives. J Stud Alcohol 1991;52:197–204. 2046369 10.15288/jsa.1991.52.197

[25] Bois C, Vogel-Sprott M Discrimination of low blood alcohol levels and self-titration skills in social drinkers. Q J Stud Alcohol 1974;35:86–97. 4827297

[26] Higgs S, Stafford LD, Attwood AS, Cues that signal the alcohol content of a beverage and their effectiveness at altering drinking rates in young social drinkers. Alcohol 2008;43:630–5. 10.1093/alcalc/agn053 18583545

[27] Saunders JB, Aasland OG, Babor TF, Development of the alcohol use disorders identification test (AUDIT). WHO collaborative project on early detection of persons with harmful alcohol consumption-II. Addiction 1993;88:791. 8329970 10.1111/j.1360-0443.1993.tb02093.x

[28] Bohn MJ, Krahn DD, Staehler BA Development and initial validation of a measure of drinking urges in abstinent alcoholics. Alcohol Clin Exp 1995;19:600–6. 10.1111/j.1530-0277.1995.tb01554.x 7573780

[29] Watson D, Clark LA, Tellegen A Development and validation of brief measures of positive and negative affect: the PANAS scales. J Pers Soc Psychol 1988;54: 1063–70. 3397865 10.1037//0022-3514.54.6.1063

[30] Licensing Act 2003 Act of UK Parliament. Available at: http://www.legislation.gov.uk/ukpga/2003/17/contents (22 July 2016, date last accessed).

[31] Van Kleef E, Kavvouris C, van Trijp HC The unit size effect of indulgent food: how eating smaller sized items signals impulsivity and makes consumers eat less. Psychol Health 2014;29:1081–103. 24678943 10.1080/08870446.2014.909426

[32] Geier AB, Rozin P, Doros G Unit bias a new heuristic that helps explain the effect of portion size on food intake. Psychol Sci 2006;17:521–5. 16771803 10.1111/j.1467-9280.2006.01738.x

[33] Nisbett RE Determinants of food intake in obesity. Science 1968;159:1254–5. 5711760 10.1126/science.159.3820.1254

[34] Robinson E, Hardman CA, Halford JC, Jones A Eating under observation: a systematic review and meta-analysis of the effect that heightened awareness of observation has on laboratory measured energy intake. Am J Clin Nutr 2015;102: 324–37. 26178730 10.3945/ajcn.115.111195

[35] Jones A, Button E, Rose AK, The ad-libitum alcohol ‘taste test’: secondary analyses of potential confounds and construct validity. Psychopharmacology 2016;233:917–24. 26680342 10.1007/s00213-015-4171-z PMC4751185

