# Supplementary material for: Effect of glass markings on drinking rate in social alcohol drinkers
Source: Eur J Public Health. 2016 Oct 20;27(2):352–6. doi: 10.1093/eurpub/ckw142 (PMC5398342; doi:10.1093/eurpub/ckw142)
Supplement: Supplementary Data [file ckw142_Supplementary_Data.zip › ejph-2016-02-om-0111-File003.doc]

**Supplementary Information**

**Table S1.** Effect of Glass Markings on Post-Consumption Alcohol Craving and Mood in Studies 1 and 2.

|  | | Adjusted for baseline | |
| --- | --- | --- | --- |
|  | | Mean difference | 95% CI |
| *Study 1* | | | |
| Full sample (n = 159) | AUQ | 0.002 | -0.26, 0.26 |
|  | Happiness | -0.87 | -4.05, 2.31 |
|  | Drowsiness | -1.45 | -7.22, 4.32 |
|  | Depression | -2.28 | -5.21, 0.66 |
|  | Anxiety | -3.42 | -7.48, 0.64 |
|  | Energy | -0.29 | -5.35, 4.77 |
|  | Irritability | -2.64 | -6.67, 1.39 |
|  | Alcohol craving | 1.08 | -4.01, 6.16 |
| Outliers excluded (n = 156) | AUQ | -0.01 | -0.27, 0.26 |
|  | Happiness | -0.60 | -3.82, 2.63 |
|  | Drowsiness | -1.61 | -7.49, 4.27 |
|  | Depression | -2.41 | -5.39, 0.58 |
|  | Anxiety | -3.05 | -7.11, 1.01 |
|  | Energy | 0.20 | -4.95, 5.36 |
|  | Irritability | -2.76 | -6.86, 1.35 |
|  | Alcohol craving | 1.32 | -3.86, 6.49 |
| *Study 2* | | | |
| Full sample (n = 160) | AUQ | 0.20 | -0.06, 0.45 |
|  | Positive affect | 0.35 | -0.90, 1.59 |
|  | Negative affect | -0.05 | -0.42, 0.33 |
| Outliers excluded (n = 156) | AUQ | 0.19 | -0.07, 0.45 |
|  | Positive affect | 0.26 | -0.99, 1.51 |
|  | Negative affect | -0.04 | -0.42, 0.34 |

^a^ AUQ = Alcohol Urges Questionnaire. ^b^ Outliers defined as 1.5 times the interquartile range above quartile 3 or below quartile 1.
